# Evaluating Locally Run Large Language Models (Gemma 2, Mistral Nemo, and Llama 3) for Outpatient Otorhinolaryngology Care: Retrospective Study

**DOI:** 10.2196/76896

**Published:** 2025-11-25

**Authors:** Christoph Raphael Buhr, Christopher Seifen, Katharina Bahr-Hamm, Tilman Huppertz, Johannes Pordzik, Harry Smith, Tom Kelsey, Andrew Blaikie, Christoph Matthias, Sebastian Kuhn, Jonas Eckrich

**Affiliations:** 1 Department of Otorhinolaryngology, University Medical Center of the Johannes Gutenberg-University Mainz, Langenbeckstraße 1, Mainz, 55131, Germany, +49 6131 17 7362; 2 School of Medicine, University of St Andrews, St Andrews, United Kingdom; 3 School of Computer Science, University of St Andrews, St Andrews, United Kingdom; 4 Institute for Digital Medicine, Philipps University Marburg, University Hospital Giessen and Marburg, Marburg, Germany

**Keywords:** large language models, artificial intelligence, otorhinolaryngology, digital health, chatbot, global health, low- and middle-income countries, telemedicine, telehealth

## Abstract

**Background:**

Large language models (LLMs) have great potential to improve and make the work of clinicians more efficient. Previous studies have mainly focused on web-based services, such as ChatGPT, often with simulated cases. For the processing of personalized patient data, web-based services have major data protection concerns. Ensuring compliance with data protection and medical device regulations therefore remains a critical challenge for adopting LLMs in clinical settings.

**Objective:**

This retrospective single-center study aimed to evaluate locally run LLMs (Gemma 2, Mistral Nemo, and Llama 3) in providing diagnosis and treatment recommendation for real-world outpatient cases in otorhinolaryngology (ORL).

**Methods:**

Outpatient cases (n=30) from regular consultation hours and the emergency service at a university hospital ORL outpatient department were randomly selected. Documentation by ORL doctors, including anamnesis and examination results, was passed to the locally run LLMs (Gemma 2, Mistral Nemo, and Llama 3), which were asked to provide diagnostic and treatment strategies. Recommendations of the LLMs and the treating ORL doctors were rated by 3 experienced ORL consultants on a 6-point Likert scale for medical adequacy, conciseness, coherence, and comprehensibility. Moreover, consultants were asked whether the answers pose a risk to the patient’s safety. A modified Turing test was performed to distinguish responses generated by LLMs from those of doctors. Finally, the potential influence of the information generated by the LLMs on the raters’ own diagnosis and treatment opinions was evaluated.

**Results:**

Over all categories, ORL doctors achieved superior (*P*<.0005) ratings compared to locally run LLMs (Llama 3, Mistral Nemo, and Gemma 2). ORL doctors’ responses were considered hazardous for patients in only 1% of the ratings, whereas recommendations by Llama 3, Gemma 2, and Mistral Nemo were considered hazardous in 54%, 47%, and 32% of cases, respectively. According to the raters, the LLM’s information rarely influenced their judgment, with Mistral Nemo, Gemma 2, and Llama 3 achieving 1%, 3%, and 4% of the ratings, respectively.

**Conclusions:**

Although locally run LLM models still underperform compared with their web-based counterparts, they achieved respectable results on outpatient treatment in this study. Nevertheless, the retrospective and single-center nature of the study, along with the clinicians’ documentation style, may have introduced bias in favor of human recommendations. In the future, locally run LLMs will help address data protection concerns; however, further refinement and prospective validation are still needed to meet strict medical device requirements. As locally run LLMs continue to evolve, they are likely to become comparably powerful to web-based LLMs and become established as useful tools to support doctors in clinical practice.

## Introduction

### Potential of Large Language Models in Medicine

The introduction of new large language models (LLMs), such as ChatGPT (OpenAI, California, USA), has disrupted the traditional perceptions of artificial intelligence (AI). Rather than requiring extensive coding skills to achieve a task, LLMs understand natural human language input, making their capabilities accessible to much broader tasks. Instead of being trained for a specific purpose, these models are ‘all-rounders’ capable of accomplishing a wide range of different tasks, including medical queries [1,2]. When required, further fine-tuning can improve their performance for certain fields of action while achieving higher cost-efficiency than training a whole new model for a specific purpose. These characteristics are essential for medical applications. First, medical data such as anamnesis and examination findings are usually recorded in a semistructured manner in human language. Second, the broadly spread competence of LLMs is helpful for understanding the complex and interlinked issues patients present to health systems. Third, with regard to increasing economic pressure in the health care system, LLM support might present a promising solution to increase efficacy, improve outcomes, and reduce costs.

### LLMs in Otorhinolaryngology

The application of LLMs in otorhinolaryngology (ORL) is the subject of current research. Suggested areas of application include research and clinical use. Clinical uses range from patient education to improving electronic medical records, triage, patient classification, clinical education, and decision support [3]. More specifically, recent studies have evaluated LLMs in answering patients’ questions [4] over the analysis of polysomnographic results [5] to tumor board augmentation [6,7]. Nevertheless, the most frequent touch points between ORL doctors and patients occur in the outpatient care sector.

Thus, this area of application offers one of the most relevant instances where support of ORL doctors by LLMs may be useful. Previous studies in this area highlighted the promising performance of LLMs, while stating the overall superiority of ORL consultants. A study from 2023 compared LLM recommendations on case-based questions with those of experienced ORL consultants. In this case, the LLMs received high ratings in semantic categories and promising ratings on medical adequacy. Furthermore, the study revealed a significant improvement in medical adequacy between 2 tested versions (ie, ChatGPT 3 and ChatGPT 4) [8]. A further, similarly designed study assessed differences between various LLMs. Here, Claude 2 (Anthropic, California, USA) and ChatGPT 4 achieved the highest ratings on medical adequacy, whereas answers from ChatGPT 4 proved to be the most secure for the patients [9]. While both studies cited earlier used rated categories as performance metrics (ie, medical adequacy, coherence, comprehensibility, and conciseness, each rated on a Likert scale), another study primarily evaluated agreement between physicians’ recommendations and those of the LLMs. In this study, the agreement of physicians and ChatGPT on physician-written clinical vignettes in otolaryngology was rated using a 5-point Likert scale. The authors showed high agreement of ChatGPT and the physicians on differential diagnosis and treatment plans, with no association between vignette difficulty and agreement with differential diagnosis or treatment [10].

### Conquering Data Protection Challenges: The Potential of Locally Run LLMs

Despite the promising findings these studies offer, they often use highly structured (and simulated) input data, mainly focusing on web-based LLMs, such as ChatGPT. This approach has obvious data protection limitations when it comes to real-world implementation. Data protection requirements are the key constraint when using web-based LLMs, as sharing highly sensitive patient data with a third party raises confidentiality concerns. While the General Data Protection Regulation [11] regulates secure data processing in the European Union, the Health Insurance Portability and Accountability Act [12] is of utmost importance in the United States. Unlike web-based LLMs, locally operated LLMs can mitigate data protection concerns by avoiding external data transfers. To date, locally run LLMs have received little attention for use in medical queries, especially for outpatient treatment, despite the potential benefits they could provide. Thus, this study evaluates different locally run open-access LLMs (ie, Llama 3 (Meta, California, USA), Mistral Nemo (Mistral AI, Paris, France), and Gemma 2 (Google, California, USA)) in providing diagnosis and therapy recommendation for real outpatient cases with no data sharing and confidentiality concerns.

## Methods

### Study Design and Patient Case Selection

**Figure 1.**
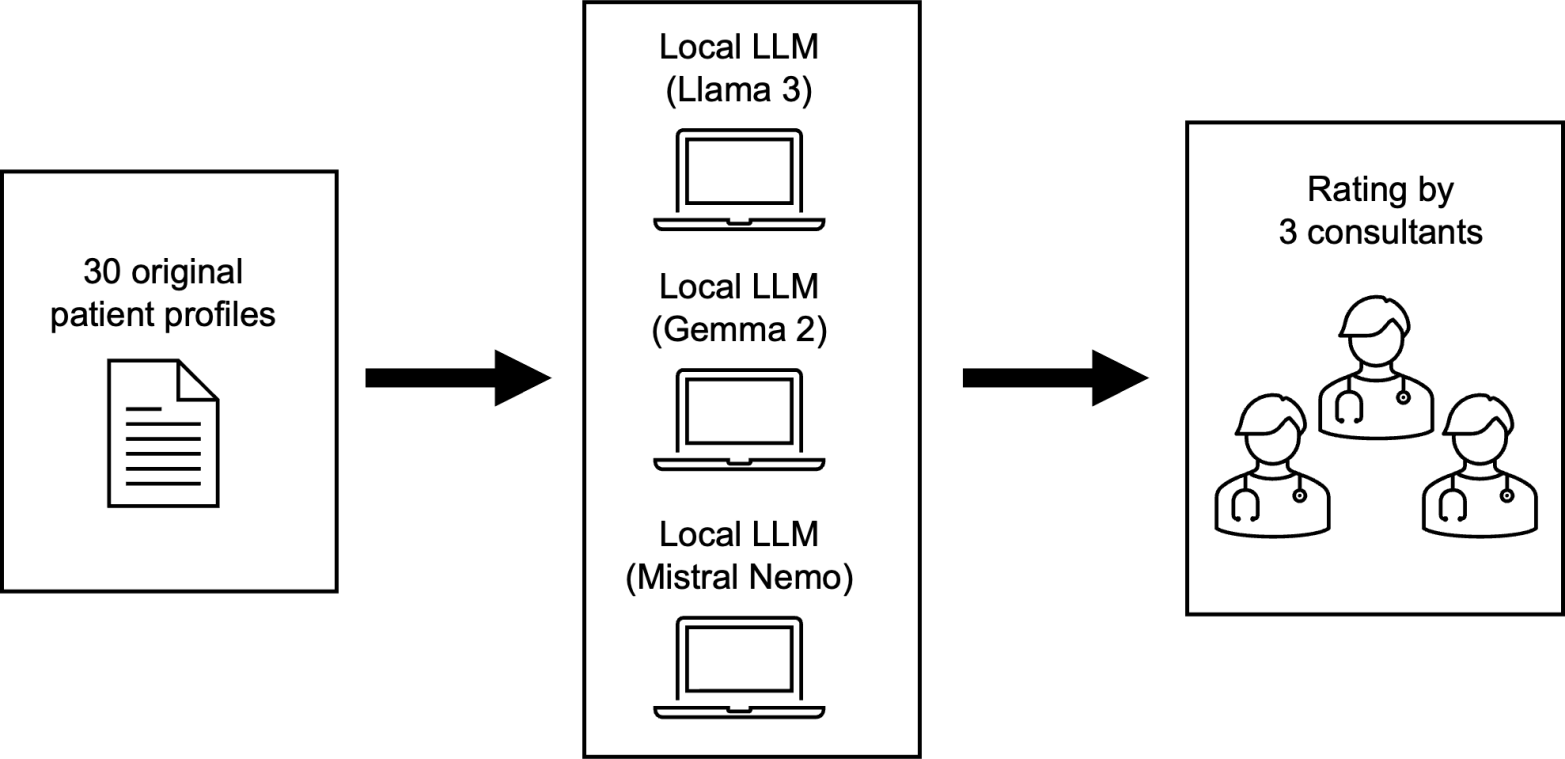
Workflow of the study. LLM: large language model.

The workflow of the study is visualized in Figure 1. Thirty outpatient cases from the archive of our regular consultation hours and the emergency service at the university hospital ORL outpatient office were retrieved for the study. Cases were selected at random from attendance lists. The selection was designed to be as broad as possible, and duplication was avoided as the frequency of different diseases differs. In case of duplication (same disease as one in the previous case), a new case was selected. The retrieved cases involved the following diagnoses: chronic sinusitis and frontal osteoma, tinnitus and conductive hearing loss on both sides, nasal polyp on the left side, deviated septum, conchal hyperplasia, functional voice disorder, dysosmia, recurrent chronic sinusitis, chronic sinusitis, median neck cyst, vocal cord/fold polyp, chronic otitis media, tympanic effusions on both sides, suspected adenoid vegetations, nasal bone fracture, nasal bridge laceration, severe sensorineural hearing loss, vallecula cyst, tumor in the parotid gland, chronic mesotympanic otitis media, lymphadenopathy, benign paroxysmal positional vertigo and vestibulopathy, tonsillar carcinoma, benign paroxysmal positional vertigo, acute bilateral tonsillitis, otitis externa, sillar abscess, foreign body esophagus, foreign body ear, epistaxis after septoplasty, obstructive ear wax, acute unilateral vestibulopathy, and otitis media.

### Data Preparation and Prompting

**Figure 2.**
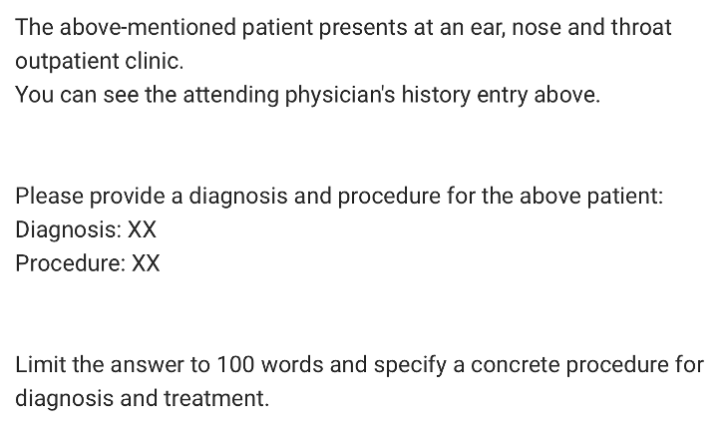
Prompt use within this study.

The selected cases were saved in a text document (Word; Microsoft, Redmond, California, USA). The entry also included the patient’s gender and year of birth. Generally, medical documentation of the ORL doctors in our clinic is subdivided into anamnesis, examination findings, diagnosis, and treatment recommendation. Therefore, the diagnoses and treatment recommendation section was removed before each patient case was passed to the locally run LLMs (ie, Llama 3, Mistal Nemo, and Gemma 2). All cases were passed to the different locally run LLMs using the basic prompt shown in Figure 2 in the German language. The prompt refers to ORL doctors’ documentation style in our clinic and explicitly requests the diagnosis and treatment recommendation, limiting the answer to 100 words. The prompt has been kept deliberately simple to test the LLM’s baseline performance. The instructions provided aimed to ensure that the response resembles the doctors’ documentation style.

### LLM Execution

The LLMs were run locally on a standard Hewlett-Packard (Palo Alto, California, USA) Notebook (Intel Core 17‐1255U, 4.7 GHz. DDR4, 16 GB [2×8 GB], Windows 10 Pro, LM Studio 0.3.5) using the prompt shown in Figure 2 as stated earlier.

The selection of LLMs was guided by their open-source availability and model size. Consequently, only models below 10 GB were included. The default settings of LLM Studio were not modified, and all model configurations— including parameter sizes or variants, quantization settings, prompt templates and system prompts, as well as temperature, top-p, maximum token limits, and random seeds—were kept unchanged. The specific models evaluated in this study are as follows: Meta-Llama-3-8B-Instruct-GGUF (GPU Offload 32/32; CPU Thread Pool Size 4, Evaluation Batch Size 512, Context Length 4096, Random Seed), Gemma-2-9b-it-GGUF (GPU Offload 37/42; CPU Thread Pool Size 4, Evaluation Batch Size 512, Context Length 4096, Random Seed), and Mistral-Nemo-Instruct-2407-GGUF (GPU Offload 27/40; CPU Thread Pool Size 4, Evaluation Batch Size 512, Context Length 4096, Random Seed) (all published by lmstudio-community).

### Evaluation and Rating

To evaluate the results, all cases were collected in a single text document. In particular, the doctor’s documentation (eg, medical history and examination findings) was presented first for each case. Subsequently, the diagnoses and treatment recommendations of the 3 LLMs and the treating ORL doctor were presented in random order for the specific case. The evaluation criteria stated below were filled in under each answer (Likert scale and binary rating) by each rater.

The recommendation of the LLMs and the original documentation of the treating ORL doctors were rated by 3 experienced ORL consultants on a 6-point Likert scale (1=very poor and 6=excellent) for medical adequacy, conciseness, coherence, and comprehensibility.

The key assessment metrics were defined as follows:

- *Medical adequacy* refers to the accuracy and appropriateness of medical content relative to established clinical guidelines and expert consensus.
- *Coherence* refers to the logical flow and consistency of the information presented. This was assessed by reviewers based on the clarity of connections between ideas and the absence of contradictory statements.
- *Comprehensibility* measures the ease with which the information can be understood.
- *Conciseness* evaluates whether the information is sufficiently succinct without omitting critical details.

Furthermore, *hazardous for patients* was defined as the presence of information that could directly or indirectly lead to patient harm if followed. This includes advice that contradicts established clinical guidelines, promotes unsafe practices, misrepresents risks or benefits, or could result in delayed diagnosis, inappropriate treatment, or adverse outcomes.

Moreover, ORL consultants were asked (binary rating) whether the answers pose a risk to the patient’s safety, whether the response originates from an LLM or a doctor (modified Turing test) [13], and whether their own diagnosis and treatment opinion would be influenced by the respective answer. In total, for each included case, the 3 experienced ORL consultants rated the response (diagnosis and treatment recommendation) of the treating ORL doctor and every tested LLM.

### Benchmarking Against Web-Based LLMs

Web-based LLMs have been previously evaluated as mentioned earlier [8,9] and have shown sufficient capabilities in evaluating case-based questions. To benchmark the locally run LLMs with an established web-based LLM, 10 simulated cases were comparatively evaluated following the same workflow during the course of the study. Accordingly, the 10 simulated cases were processed by an ORL doctor and submitted to the 3 locally run LLMs (ie, Llama 3, Mistal Nemo, and Gemma 2), as well as a web-based LLM (ie, Chat GPT-4o).

### Statistical Analysis

All responses from the raters were transferred to a Microsoft Excel spreadsheet and sorted according to the entity being evaluated. Statistical analysis was performed with GraphPad Prism for macOS (version 10.3.0; GraphPad Software, La Jolla, CA, USA). The data did not meet normality assumptions, as confirmed by the D’Agostino and Pearson test.

Further statistical analysis was performed using Python (version 3.11.13) in the Google Colab environment. The data were imported from Excel files. Packages used include pandas (data processing), statsmodels, and pingouin.

For each rating category (ie, medical adequacy, conciseness, coherence, and comprehensibility), a linear mixed effects model was fitted, with entity as a fixed effect and case and rater as random intercepts to account for clustering within cases and raters. Furthermore, interrater reliability was examined using evaluations of the same responses by multiple raters.

The following measures were used to calculate reliability:

- Fleiss κ, a measure of agreement in categorical evaluations, was determined using the fleiss_kappa function from the statsmodels package.
- The percentage agreement was calculated as the proportion of cases in which all raters gave exactly the same rating.
- The intraclass correlation coefficient (ICC) for quantifying agreement in ordinal ratings was calculated using the intraclass_corr function from the pingouin package. For this purpose, the data were transformed into a long format containing target objects, raters, and ratings.

### Ethical Considerations

Ethical approval was obtained from the ethics committee of the state medical association (request/approval number: 2023‐17385-retrospektiv). Informed consent was not required as this was a retrospective study. The data were processed anonymously to ensure privacy, and compensation was not necessary due to the retrospective nature of the study.

## Results

### Rating Results

**Figure 3.**
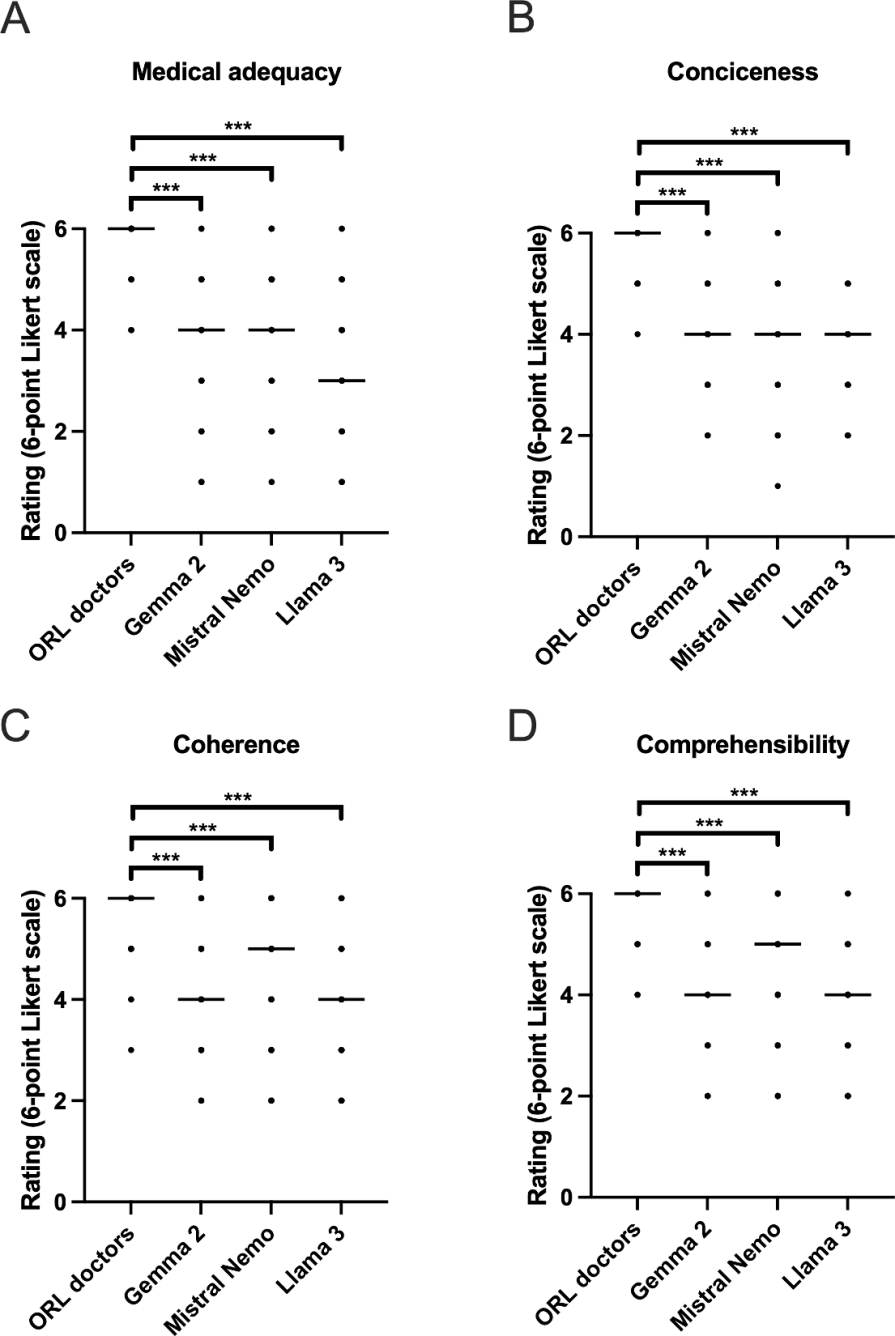
Rating for locally run LLMs and ORL consultants on providing diagnosis and treatment strategy (30 real-world cases) regarding (A) medical adequacy, (B) conciseness, (C) coherence, and (D) comprehensibility rated on a 6-point Likert scale. Normality distribution was tested with the D’Agostino and Pearson test. Comparisons were performed using a linear mixed-effects model. Nonsignificant >0.05, *P<.05, **P<.005, ***P<.001. LLM: large language models; ORL: otorhinolaryngology.

ORL doctors showed significantly (*P*<.05%) higher ratings compared to the locally run LLMs (ie, Llama 3, Mistral Nemo, and Gemma 2) over all categories (Figure 3). Altogether, the doctors achieved (median 6, interquartile range [IQR] 5‐6) the highest rating in every category. On medical adequacy, Gemma 2 and Mistral Nemo received similar ratings (Mistral Nemo: median 4, IQR 3-5; and Gemma: median 4, IQR 3-4), whereas Llama 3 performed inferior scores, achieving a median of 3 (IQR 3-4). For conciseness, all locally run LLMs tested received similar ratings (Gemma 2 and Mistral Nemo: median 4, IQR 4-5; and Llama 3: median 4, IQR 3-4). Mistral Nemo (median 5, IQR 4-5) outperformed Gemma 2 and Llama 3 on ratings for coherence and comprehensibility. Both Gemma 2 and Llama 3 received similar ratings in these categories. Detailed rating results are presented in Table 1. Details of the linear mixed effects model for predicting ratings are presented in Table 2, and *P* values for entity coefficients were all <.001, indicating a significant difference between ORL doctors and LLMs. Boxplots of the ratings categorized for each model can be found in Multimedia Appendix 1 .

**Table 1.** Results of the rating by ORL^a^ consultants.

	Values, mean (SD)	Rating, median (IQR^b^)	95% CI of median
**Medical adequacy**			
ORL doctors	5.6 (0.6)	6 (5-6)	6-6
Gemma 2	3.5 (1.2)	4 (3-4)	3-4
Mistral Nemo	4 (1.2)	4 (3-5)	4-4
Llama 3	3.4 (1.2)	3 (3-4)	3-4
**Conciseness**			
ORL doctors	5.7 (0.5)	6 (5-6)	6-6
Gemma 2	4.2 (0.9)	4 (4-5)	4-4
Mistral Nemo	4.3 (1)	4 (4-5)	4-5
Llama 3	3.8 (0.8)	4 (3-4)	4-4
**Coherence**			
ORL doctors	5.7 (0.6)	6 (5-6)	6-6
Gemma 2	4.2 (1.1)	4 (3-5)	4-5
Mistral Nemo	4.4 (1.2)	5 (4-5)	5-5
Llama 3	3.8 (1.1)	4 (3-5)	4-4
**Comprehensibility**			
ORL doctors	5.7 (0.5)	6 (6)	6-6
Gemma 2	4.3 (1)	4 (4-5)	4-5
Mistral Nemo	4.5 (1.1)	5 (4-5)	4-5
Llama 3	3.9 (1)	4 (3-5)	4-4

a ORL: otorhinolaryngology.

b IQR: interquartile range.

**Table 2.** Linear mixed effects model for ratings.

	Estimate	*P* value	ci_lower	ci_upper
**Medical adequacy**
Intercept	5.633	0	5.370	5.896
entity[T.Gemma 2]	−2.1	<.001	−2.385	−1.814
entity[T.Mistral Nemo]	−1.633	<.001	−1.918	−1.348
entity[T.Llama 3]	−2.278	<.001	−2.563	−1.993
Group Var	0.223	.01	0.045	0.402
rater Var	0.033	.517	−0.067	0.134
**Conciseness**				
Intercept	5.711	0	5.524	5.899
entity[T.Gemma 2]	−1.556	<.001	−1.773	−1.338
entity [T.Mistral Nemo]	−1.456	<.001	−1.673	−1.238
entity[T.Llama 3]	−1.922	<.001	−2.14	−1.705
Group Var	0.106	.127	−0.03	0.242
rater Var	0.164	.05	<0.001	0.329
**Coherence**				
Intercept	5.677	0	5.469	5.886
entity[T.Gemma 2]	−1.5	<.001	−1.768	−1.232
entity[T.Mistral Nemo]	−1.244	<.001	−1.512	−0.976
entity[T.Llama 3]	−1.844	<.001	−2.112	−1.576
Group Var	0.014	.716	−0.064	0.092
rater Var	0.166	.08	−0.021	0.352
**Comprehensibility**				
Intercept	5.744	0	5.526	5.963
entity[T.Gemma 2]	−1.422	<.001	−1.670	−1.174
entity[T.Mistral Nemo]	−1.267	<.001	−1.515	−1.019
entity[T.Llama 3]	−1.856	<.001	−2.104	−1.607
Group Var	0.121	.109	−0.027	0.268
rater Var	0.183	.036	0.012	0.355

### Modified Turing Test

Regarding the modified Turing test, Gemma 2 was recognized as a machine in 99% (89/90), Mistral Nemo in 98% (88/90), and Llama 3 in 97% (87/90) of the ratings. Conversely, the ORL doctors were recognized as a human being in 99% (89/90) of the ratings.

### Assessment of Patient Safety

While the raters considered the ORL doctors’ recommendations as potentially unsafe for the patient in only 1% (1/90) of cases, Llama 3’s recommendations were considered potentially hazardous in 54% (49/90), Gemma 2’s in 47% (42/90), and Mistral Nemo’s in 32% (29/90) of cases.

### Influence on Raters’ Own Decision-Making

Whereas the ORL consultants stated that the information provided by the ORL doctors might have influenced their own decision in 2% (2/90), Mistral Nemo was considered influential in 1% (1/90), Gemma 2 in 3% (3/90), and Llama 3 in 4% (4/90) of cases for this assessment.

### Number of Words

**Figure 4.**
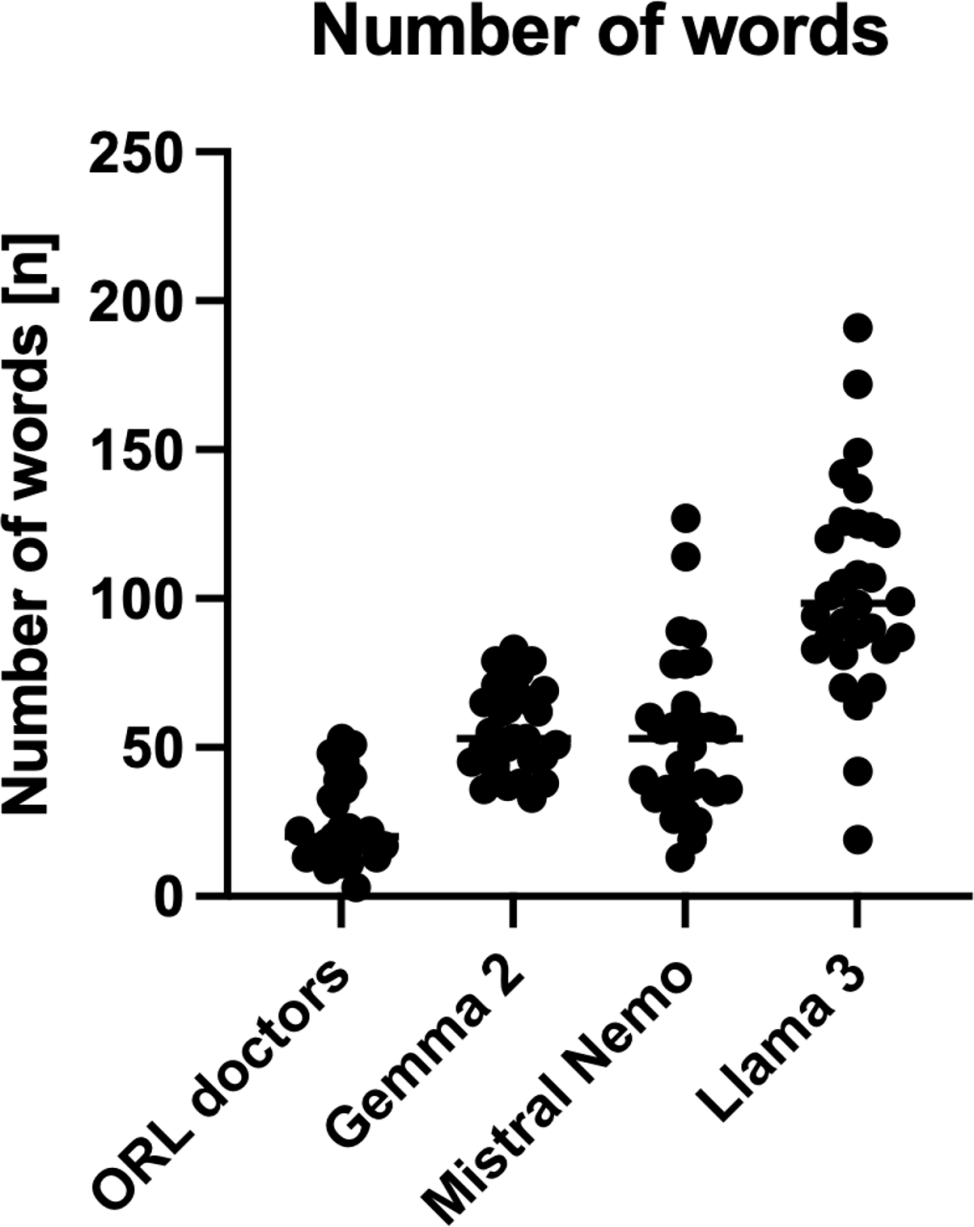
Number of words used by ORL doctors and the locally run LLMs (ie, Gemma 2, Mistral Nemo, and Llama 3) on providing diagnosis and treatment recommendations.

The number of words used by the ORL doctors and the locally run LLMs is visualized in Figure 4. Llama 3 (median 99, range 19‐191, IQR 83‐124) spent the most words on providing a diagnosis and treatment recommendations, followed by Gemma 2 (median 53, range 33‐83, IQR 45‐68) and Mistral Nemo (median 53, range 13‐127, IQR 35‐68) and the ORL doctors (median 20, range 3‐53, IQR 13‐37).

### Interrater Reliability

The interrater reliability analysis using Fleiss κ and percentage match is presented in Table 3. A further interrater reliability analysis applying the ICC is illustrated in Table 3.

**Table 3.** Overall interrater reliability: Fleiss κ and percentage match.

Category	Fleiss κ^a^ Rater 1‐3 170 responses evaluated	Percentage of homolog ratings between all raters^b^: $\frac{number\;of\;homolog\;ratings\;between\;all\;raters}{number\;of\;cases}$
Modified turing test	0.906	0.947
Hazardous for patients	0.419	0.635
Influence by the respective answer	−0.028	0.917
Medical adequacy	0.241	0.206
Conciseness	0.167	0.165
Coherence	0.152	0.159
Comprehensibility	0.186	0.188

a Interpretation of Cohen’s κ adapted from Landis and Koch (1977).

b <0: worse than chance; 0.00-0.20: low agreement; 0.21-0.40: moderate agreement; 0.41-0.60: satisfactory correlation; 0.61-0.80: good correlation; 0.81-1.00: very good to perfect correlation.

## Discussion

### Context of the study

Previous studies assessing LLMs on outpatient treatment in ORL focused on web-based LLMs mainly using simulated cases [8-10,14]. While web-based LLMs show impressive linguistic and professional capabilities, this setup has obvious limitations due to data sharing concerns. These concerns could be circumvented using LLMs that have no connection to the internet working on a local computer. Hence, this study evaluates the capabilities of 3 different locally run LLMs (ie, Llama 3, Mistral Nemo, and Gemma 2) on real patient data from outpatient consultations of a University Medical Center in Germany.

Despite ORL doctors receiving superior ratings over all categories, this study demonstrates the ability of locally run LLMs to process outpatient data and provide useful diagnostic and treatment information. Regarding medical adequacy, Gemma 2 and Mistral Nemo received similar high ratings (Mistral Nemo: median 4, IQR 3-5; and Gemma: median 4, IQR 3-4), whereas Llama 3 performed inferiorly, achieving a median of 3 (IQR 3-4). Comparing with previous studies, the performance of Gemma 2 and Mistral Nemo is similar to ChatGPT 3’s performance in a study from 2023 reaching a median rating of 4 (IQR 4-5) on medical adequacy [8]. However, a comparison with a more recent study reveals an inferior rating on medical adequacy. In the mentioned study from 2024, the web-based LLMs Claude 2 (median 5, IQR 4-6), ChatGPT 4 (median 5, IQR 4-6), and Bard 2023.07.13 (median 5, IQR 3-5) achieved higher ratings on medical adequacy than the best performing locally run LLMs (ie, Mistral Nemo and Gemma 2) in this study [9]. Regarding conciseness, all locally run LLMs received similar ratings (Gemma 2 and Mistral Nemo: median 4, IQR 4-5; and Llama 3: median 4, IQR 3-4). These performance levels correspond with the results from prior studies [8,9]. Mistral Nemo (median 5, IQR 4-5) outperformed Gemma 2 and Llama 3 on ratings for coherence and comprehensibility, which received similar ratings in these categories. With regard to the modified Turing test, the ORL doctors were recognized as human beings in 99% (89/90) of the ratings, whereas the LLMs were almost always correctly identified as machines. Gemma 2 was recognized as a machine in 99% (89/90), Mistral Nemo in 98% (88/90), and Llama 3 in 97% (87/90) of the ratings. Again, these ratings from this study for coherence and comprehensibility are in line with results from previous studies [8,9].

### Hazardous for Patients

It is important to examine the cases marked as hazardous in more detail to better understand the risks posed by LLMs. The only risk identified by the treating doctor is in the case of a patient with a large nasal polyp, for whom the polyp’s removal under local anesthesia was recommended. The reason why this was assessed as a potential risk to the patient remains unclear, but it may be explained by the fact that the patient is taking anticoagulants (acetylsalicylic acid and clopidogrel). Overall, Llama 3’s recommendations were considered potentially hazardous in 54% (49/90), Gemma 2’s in 47% (42/90), and Mistral Nemo’s in 32% (29/90) of cases. A typical example of an LLM shortcoming is a case involving a patient with chronic sinusitis who reports being largely symptom-free while undergoing local cortisone therapy. While the doctor and Mistral Nemo recommend continuing with the cortisone therapy alone, Llama 3 and Gemma 2 recommend adding antibiotics to the treatment. In this case, the patient would be prescribed antibiotics unnecessarily, posing a risk to the patient, particularly given that the examination findings do not mention any pus. In another case involving a 3-year-old girl with suspected adenoids and tympanic fluid, Gemma 2 recommends myringoplasty, Mistral Nemo recommends a wait-and-see approach, and Llama 3 recommends speech therapy and a tonsillectomy. In contrast, the doctor recommends an adenotomy and the insertion of tympanic drains. Recommending the wrong operation is certainly one of the most extreme misjudgments. This demonstrates the importance of careful examination and shows that, despite their eloquence, LLMs should not be trusted unconditionally.

### Benchmarking With Web-Based LLMs

The comparison with preceding underscores reveals the improvement of LLMs over time [8,9]. To benchmark the locally run LLMs with their current web-based counterparts, an assessment on 10 separate simulated cases (simulated to meet data protection constraints) was performed. Here, the web-based LLM ChatGPT-4o and ORL doctors achieved similar high ratings on medical adequacy (ORL doctors: median 6, IQR 6; ChatGPT-4o: median 6, IQR 5-6). Regarding patients’ security, a potential hazard for patients was noted in a single rating for ChatGPT-4o (1/30, 3%), in 53% (16/30) for Llama 3, and in 23% (7/30) for Mistral Nemo and Gemma 2. None of the ORL doctors’ recommendations was considered as potentially hazardous for patients. In addition, the raters found ChatGPT-4o to potentially influence their own judgment in 10% (3/30) of the cases. Among the locally run LLMs, only Llama 3 received a single positive rating in this dimension (3%; detailed data regarding the web-based LLM assessment are present in Multimedia Appendix 1). Interestingly, ChatGPT-4o has only been accused of jeopardizing a patient in one case. In this case, the patient had a suspicious cervical lymph node, and the doctor recommended extirpation. However, ChatGPT-4o recommended fine needle aspiration, laboratory tests, a magnetic resonance imaging scan, and a follow-up appointment. The reason for flagging this answer as risky is probably that the LLM did not commit to any procedure. Mistral Nemo recommended a biopsy or excision, Gemma recommended a biopsy and a blood test, and Llama suggested a check-up in 4 weeks. Although the assessment of the web-based LLMs did only include 10 simulated cases rather than real-world data, it does demonstrate the high-performance level of current web-based LLMs and their leading edge over the tested locally run competitors.

Recent developments in advanced reasoning LLMs (often referred to as chain-of-thought or system-prompt–augmented models) could further improve diagnostic accuracy and reduce risky recommendations by simulating more nuanced clinical reasoning processes. These models leverage step-by-step logical reasoning, potentially enabling them to identify and correct errors before delivering final outputs.

### Interrater Reliability

Analyzing interrater reliability can be challenging. In this study, the evaluating ORL consultants demonstrated an excellent Fleiss κ correlation of 0.906 in the modified Turing test (with binary answers). For the assessment of hazards to patients (also binary answers), a Fleiss κ of 0.419 was found, indicating a satisfactory level of correlation. Furthermore, ORL consultants were asked whether the presented answer would have influenced their own decision (binary rating). This produced a Fleiss κ of −0.028, which formally corresponds to poorer correlation than expected by chance. However, assessment of the raw data showed high concordance between raters; therefore, we include the percentage of homolog ratings between all raters, which was 91.7% (number of homolog ratings between all raters/number of cases). Taking these measures into consideration, the low Fleiss κ value is therefore most likely caused by the high uniformity of the ratings provided.

Fleiss κ and the percentage of homolog ratings between all raters suggested low interrater reliability for ratings in the categories of medical adequacy, conciseness, coherence, and comprehensibility. The same applied for the absolute agreement. However, an examination of the raw data revealed that the raters’ assessments were in fact highly consistent throughout. Although the raters often did not award exactly the same points, the scores awarded were close to each other, for example, rating 5 points and rating 6 points for the same answer by different raters. Hence, we calculated ICCs. The ICC analysis shows a moderate (ICC≈0.46‐0.65) interrater agreement in the abovementioned categories (Table 4). The aggregated assessment of several raters (ICCk) achieved moderate to good values in all categories (ICCk ≈0.72‐0.85). The highest agreement was for medical adequacy, the lowest for comprehensibility. All ICC values were highly significant (*P*<.001), with narrow CIs, indicating stable estimates. In conclusion, despite the fact that all rating ORL consultants rarely choose exactly the same rating for the responses given, they highly agreed within the tendency of ratings (Table 4). Overall, this analysis reveals a high consistency among ratings.

**Table 4.** Overall interrater reliability: intraclass correlation coefficient (ICC).

Category	Description	ICC	*F* statistic	df1^a^	df2^a^	*P* value	95% CI^b^
**Medical adequacy**							
ICC1^c^	Single raters absolute	0.653	6.643	169	340	<.001	0.58-0.72
ICC2^d^	Single random raters	0.653	6.614	169	338	<.001	0.58-0.72
ICC3^e^	Single fixed raters	0.652	6.614	169	338	<.001	0.58-0.72
ICC1k^f^	Average raters absolute	0.849	6.643	169	340	<.001	0.81-0.88
ICC2k^f^	Average random raters	0.849	6.614	169	338	<.001	0.81-0.88
ICC3k^f^	Average fixed raters	0.849	6.614	169	338	<.001	0.8-0.88
**Conciseness**							
ICC1	Single raters absolute	0.544	4.575	169	340	<.001	0.46-0.62
ICC2	Single random raters	0.552	5.221	169	338	<.001	0.45-0.64
ICC3	Single fixed raters	0.585	5.221	169	338	<.001	0.5-0.66
ICC1k	Average raters absolute	0.781	4.575	169	340	<.001	0.72, 0.83
ICC2k	Average random raters	0.787	5.221	169	338	<.001	0.71-0.84
ICC3k	Average fixed raters	0.808	5.221	169	338	<.001	0.75-0.85
**Coherence**							
ICC1	Single raters absolute	0.500	4.000	169	340	<.001	0.41-0.58
ICC2	Single random raters	0.512	4.680	169	338	<.001	0.4-0.61
ICC3	Single fixed raters	0.551	4.680	169	338	<.001	0.47-0.63
ICC1k	Average raters absolute	0.750	4.000	169	340	<.001	0.68-0.81
ICC2k	Average random raters	0.759	4.680	169	338	<.001	0.66-0.83
ICC3k	Average fixed raters	0.786	4.680	169	338	<.001	0.72-0.84
**Comprehensibility**							
ICC1	Single raters absolute	0.457	3.523	169	340	<.001	0.37-0.55
ICC2	Single random raters	0.478	4.524	169	338	<.001	0.33-0.6
ICC3	Single fixed raters	0.540	4.524	169	338	<.001	0.45-0.62
ICC1k	Average raters absolute	0.716	3.523	169	340	<.001	0.63-0.78
ICC2k	Average random raters	0.733	4.524	169	338	<.001	0.59-0.82
ICC3k	Average fixed raters	0.779	4.524	169	338	<.001	0.71-0.83

a df1, df2: degrees of freedom.

b Interpretation guidelines (Koo & Li, 2016) [15]: <0.50, poor; 0.50‐0.75, moderate; 0.75‐0.90, good; >0.90, excellent.

c ICC1: single-rater, 1-way random effects (absolute agreement).

d ICC2: single-rater, 2-way random effects (absolute agreement).

e ICC3: single-rater, 2-way mixed effects (absolute agreement).

f ICC1k, ICC2k, ICC3k: corresponding average measures ICCs.

### Limitations

Passing original text documentation to the LLMs is both a strength and a limitation of our study at the same time. Original text input in the local language (German in this study) represents the most realistic application in clinical practice. Despite the advantage of a realistic setup, the use of the German language may have had a negative impact on the results. This is indicated by the fact that some LLMs occasionally responded in English, although the rest of the context was in German. Furthermore, ORL doctors’ original documentation included grammatical errors and misspellings. Moreover, the documentation provided was limited to the text transcript of a single consultation, not including further diagnostic information, such as imaging diagnostic results. This approach was determined by the input structure of the locally run LLMs assessed in this study. The documentation of the treating ORL doctors may have been created on the basis of further information. Information that the ORL doctors may not include in their text documentation owing to the availability in the clinic’s documentation system. However, as the rating ORL consultants had only access to the same documentation as the locally run LLMs, a possible bias is limited.

Furthermore, documentation style between treating units may vary; the ORL doctors as well as the rating ORL consultants are affiliated with the same institution and thus may prefer a special design of documentation. This important advantage in favor of ORL doctors should be taken into account when interpreting the findings. Nonetheless, this limitation is because of the nature of single-center setting, which limits the generalizability of findings to other institutions and patient populations.

Moreover, clinicians’ own documentation style—crafted primarily for human readability—may inadvertently favor human reviewers, who are already accustomed to reading similar clinical notes, rather than locally run LLMs. This could introduce a systematic bias when comparing human- versus model-generated recommendations.

Overall, regarding documentation style, this study design applied the most realistic scenario using original documentation of treating ORL doctors. Ultimately, any other strategy (ie, adjustment of documentation) would have introduced a new bias. In addition, when implemented in clinical practice, LLMs must be evaluated using original documentation in any case.

Assessing “conciseness” poses its own challenges: the measured output is strongly influenced by how prompts are written (eg, whether specific length caps or “be concise” cues are used). This study aimed to assess the conciseness of the evaluated LLMs while limiting the word count to 100 within the prompt. Despite the narrow limits given, differences emerged. While the doctors and Gemma 2 adhered to the word limit every time, Mistral Nemo exceeded it twice, and Llama 3 exceeded it 14 times. However, future studies should evaluate standardized prompt instructions, clearly defining output constraints, and using consistent methods (eg, information density assessments or rubrics balancing completeness and brevity) to more reliably measure and compare conciseness across different models and scenarios.

Besides, the model size of locally run LLMs was limited by the computing capacity of the laptop used in this study. More powerful machines using larger locally run LLMs may achieve better results. Yet, the laptop used is a standard laptop, which appears realistic for use in clinical practice. This was in line with the aim of the study to test the current performance with the technical possibilities currently available. Moreover, implementing local LLMs on doctors’ local computers at their workstations has advantages over using large LLMs on the clinic’s own servers. First, there is no need to purchase additional computing capacity, as the computers already exist and are available, which saves costs. Furthermore, operation is still possible even if there is no connection to the central server, which makes operation significantly more robust and crisis-proof. Owing to continuous technical progress, it is to be expected that the performance of the comparatively small LLMs tested in this study will continue to increase and will surpass the performance of currently available large models in the near future. Accordingly, web-based LLMs can provide a glimpse into the future of what “small” local LLMs will be capable of in the future. In this study, this assessment was covered by benchmarking web-based LLMs on 10 simulated cases. With regard to rating capacity of the ORL consultants, this study was limited to 30 original cases from our outpatient department, causing constraints in the statistical analysis. Furthermore, the limitation to 30 original cases, duplicates in diagnoses were excluded from the random sampling of cases, which could have induced a selection bias.

Finally, the retrospective nature of this study prevents real-time validation of diagnoses and treatment recommendations. To further assess the true capabilities of local LLMs, future studies should assess prospective multicenter designs.

### Local LLM Conquer Data Protection Challenges

The utilization of LLMs in medical treatment has obvious medicolegal and ethical constraints. While locally run LLMs mitigate data protection concerns by avoiding external data transfer, compliance with regulations such as the General Data Protection Regulation [11] in the European Union or the Health Insurance Portability and Accountability Act [12] in the United States remains paramount. Organizations adopting such solutions must ensure that data handling procedures—including encryption, secure storage, and privacy-preserving protocols—are implemented to meet these regulatory standards. Moreover, establishing robust internal governance, maintaining transparency around how patient data are used, and conducting regular audits are essential for preserving patient privacy and upholding trust in AI-assisted care.

### Lingering Challenges for Clinical Integration

Beyond data protection, LLMs used for clinical decision-making may also fall under medical device regulations (eg, European Union Medical Device Regulation [16] and Food and Drug Administration guidelines in the United States [17]). The dynamic nature of LLMs, their propensity for generating unpredictable responses (so-called “hallucinations”), and the difficulty of verifying their outputs pose unique challenges in fulfilling strict validation and safety requirements. As such, developers and health care institutions must carefully assess whether the LLM’s intended use qualifies it as a regulated medical device and, if so, seek the necessary certification or approvals before deploying these technologies at scale.

However, recent studies also showed patients’ skepticism regarding AIs' decision-making even when supervised by human specialists [18,19]. These aspects illustrate that many obstacles beyond medical correctness and accuracy remain. These issues should be carefully addressed before an implementation of LLMs into clinical workflows is considered.

### Potential of a Clinical Integration

Despite these obvious limitations, this study highlights the broad ability of locally run LLMs to process medical data in ORL and provide diagnostic and treatment strategies without further fine-tuning or training. This is an important finding as LLMs are not trained for a specific narrow purpose, yet they are able to perform satisfactorily in a niche medical subspecialty, such as ORL. LLMs therefore represent a potentially highly cost-efficient means to scale diagnostic support particularly relevant to low-resource settings and financially constrained public health services. While the locally run LLMs failed to meet the high benchmark of ORL doctors, they still provide structured output, which in many cases did provide a suitable treatment strategy for the respective patients. Obviously, locally run LLMs are far from replacing human doctors in health care, especially when considering the relatively high rate of hazardous recommendations of some locally run LLMs. However, their web-based counterparts are capable of providing doctor-like answers with very few mistakes and hazardous potential. As technological evaluation continues, it is likely that locally run LLMs will catch up to the performance of their web-based counterparts. While the medicolegal and ethical aspects of an implementation of LLMs in clinical practices remain, they provide a promising solution to support medical staff. In a time of chronic shortage of trained health care workers all over the world, the feasibility of LLM use should be considered. Auspicious applications range from the assistance of local health workers in low-resource settings to triage or background support in high-resource settings. A roadmap of an implementation in clinical practice should include further validation of locally run LLMs in larger prospective studies. Besides evaluation by scientists, legislators need to take action. Current regulations such as device regulations show shortcomings for new software-based medical tools, such as LLMs. For instance, fast-track approval mechanisms for minor updates are needed to ensure that relevant updates do not delay by undergoing an entire approval process. Finally, the implementation of LLMs in clinical practice should be a joint project involving all stakeholders, including patients, manufacturers, physicians/scientists, and authorities.

### Conclusions

Considering the generalist nature of locally run LLMs evaluated in this study, their performance in this specific medical field is impressive. However, locally run LLMs are still outperformed by their web-based counterparts and human specialists. In time, however, locally run LLMs are likely to become as powerful as their current web-based equivalents, creating the realistic prospect of practical enhancement of day-to-day clinical diagnostics in both high- and low-resource settings. As only locally run LLMs meet the high data protection requirements for medical application, future studies should monitor and evaluate their performance on a larger scale, ideally within prospective multicenter clinical studies.

## Supplementary material

10.2196/76896 Multimedia Appendix 1 Boxplots illustrating ratings categorized for each model and detailed data on the web-based LLM assessment.

## Abbreviations

AI: artificial intelligence
LMM: large language models
ORL: otorhinolaryngology
