# Supplementary material for: Evaluating Locally Run Large Language Models (Gemma 2, Mistral Nemo, and Llama 3) for Outpatient Otorhinolaryngology Care: Retrospective Study
Source: JMIR Form Res. 2025 Nov 25;9:e76896. doi: 10.2196/76896 (PMC12646549; doi:10.2196/76896)
Supplement: Multimedia Appendix 1 [file formative-v9-e76896-s001.docx]

## **Supplementary Material**


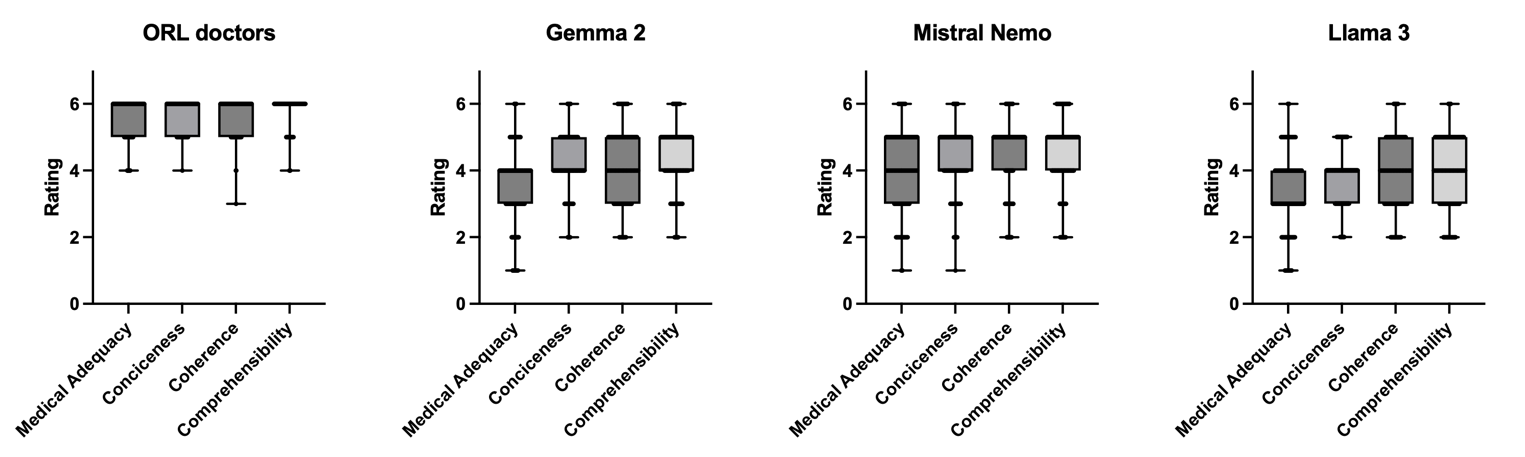


Boxplots of the ratings categorized for each model (real-world cases).

**
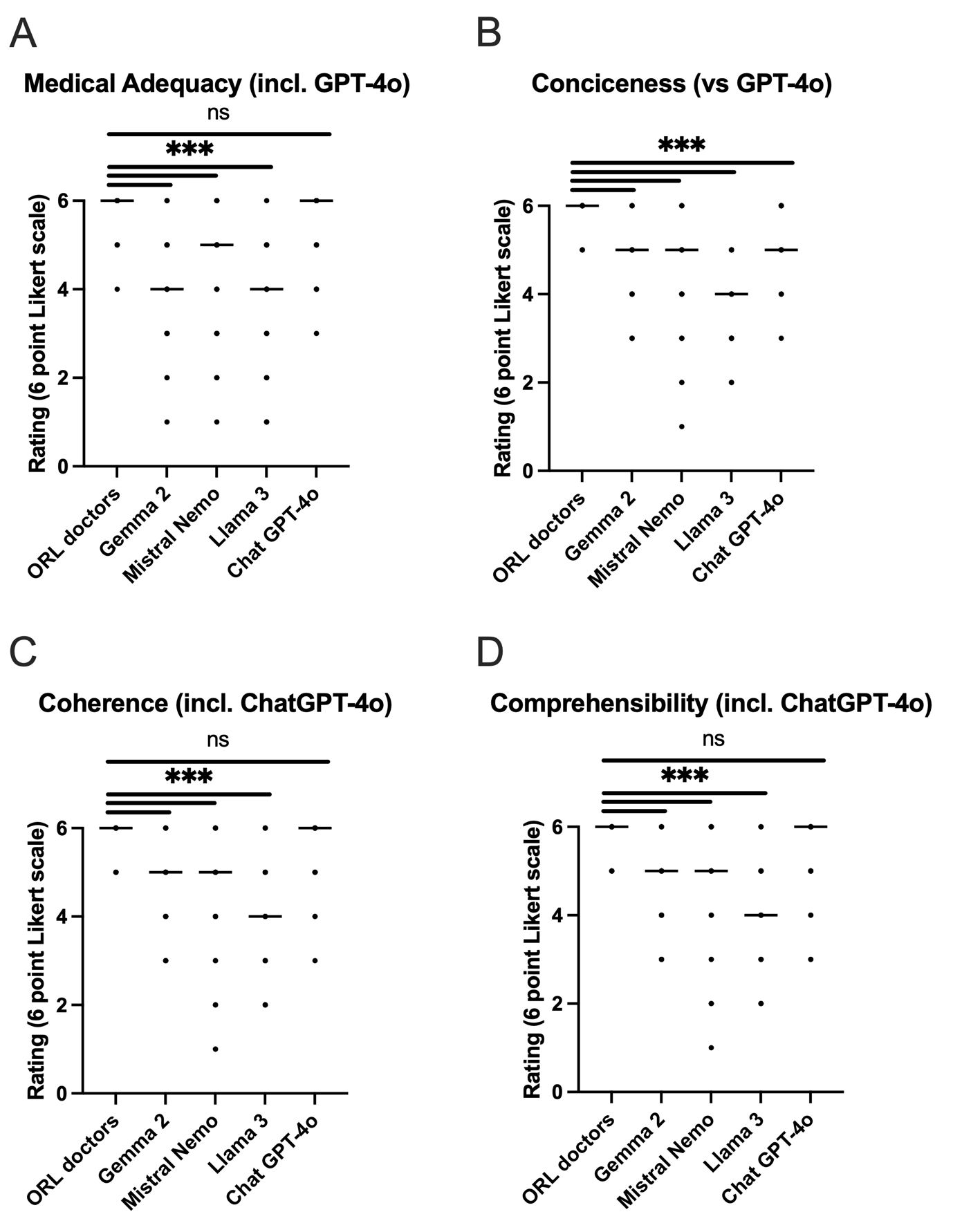
**

Ratings for simulated cases.

**
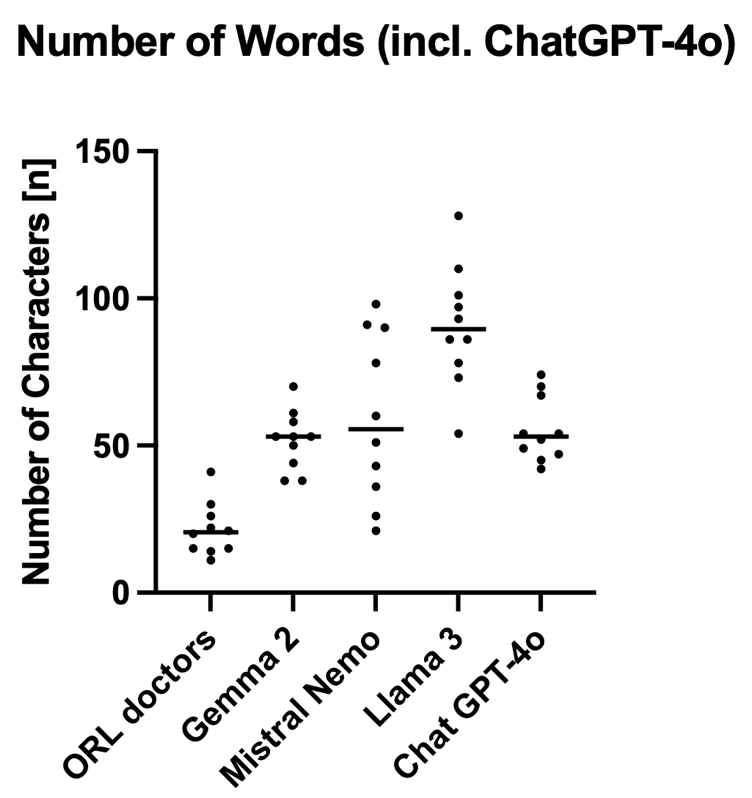
**

Number of words for simulated cases.

Linear mixed-effects model for simulated cases

| **term** | **estimate** | **p_value** | **ci_lower** | **ci_upper** | **category** |
| --- | --- | --- | --- | --- | --- |
| Intercept | 5,8 | 2,6E-119 | 5,310501 | 6,289499 | medical_adequacy |
| entity[T.Gemma 2] | -1,86667 | 1,5E-12 | -2,38383 | -1,3495 | medical_adequacy |
| entity[T.Mistral Nemo] | -1,63333 | 6,01E-10 | -2,1505 | -1,11617 | medical_adequacy |
| entity[T.Llama 3] | -2,26667 | 8,67E-18 | -2,78383 | -1,7495 | medical_adequacy |
| entity[T.ChatGPT-4o] | -0,43333 | 0,100532 | -0,9505 | 0,083834 | medical_adequacy |
| Group Var | 0,263331 | 0,098874 | -0,04941 | 0,576072 | medical_adequacy |
| rater Var | 0,001732 | 0,986102 | -0,19316 | 0,196628 | medical_adequacy |
| Intercept | 5,866667 | 4,7E-224 | 5,506818 | 6,226515 | conciseness |
| entity[T.Gemma 2] | -1,3 | 2,44E-10 | -1,70247 | -0,89753 | conciseness |
| entity[T.Mistral Nemo] | -1,53333 | 8,19E-14 | -1,9358 | -1,13087 | conciseness |
| entity[T.Llama 3] | -2,1 | 1,5E-24 | -2,50247 | -1,69753 | conciseness |
| entity[T.ChatGPT-4o] | -0,73333 | 0,000355 | -1,1358 | -0,33087 | conciseness |
| Group Var | 0,115822 | 0,472094 | -0,19988 | 0,431522 | conciseness |
| rater Var | 0,251393 | 0,267969 | -0,19341 | 0,696195 | conciseness |
| Intercept | 5,733333 | 8,7E-144 | 5,293209 | 6,173458 | coherence |
| entity[T.Gemma 2] | -1,13333 | 3,34E-06 | -1,61119 | -0,65548 | coherence |
| entity[T.Mistral Nemo] | -1,46667 | 1,79E-09 | -1,94452 | -0,98881 | coherence |
| entity[T.Llama 3] | -1,76667 | 4,28E-13 | -2,24452 | -1,28881 | coherence |
| entity[T.ChatGPT-4o] | -0,23333 | 0,338542 | -0,71119 | 0,244524 | coherence |
| Group Var | 0,179244 | 0,225144 | -0,11039 | 0,46888 | coherence |
| rater Var | 0,158893 | 0,196 | -0,08196 | 0,399746 | coherence |
| Intercept | 5,766667 | 1,9E-165 | 5,35437 | 6,178963 | comprehensibility |
| entity[T.Gemma 2] | -1,13333 | 2,02E-06 | -1,60087 | -0,66579 | comprehensibility |
| entity[T.Mistral Nemo] | -1,16667 | 1E-06 | -1,63421 | -0,69913 | comprehensibility |
| entity[T.Llama 3] | -1,63333 | 7,53E-12 | -2,10087 | -1,16579 | comprehensibility |
| entity[T.ChatGPT-4o] | -0,43333 | 0,069278 | -0,90087 | 0,034206 | comprehensibility |
| Group Var | 0,093686 | 0,582061 | -0,23995 | 0,427319 | comprehensibility |
| rater Var | 0,274239 | 0,276485 | -0,21968 | 0,768159 | comprehensibility |
